# A Meta-Analysis of the Effects of Increased Planting Density on Maize Yield in Northeast China

**DOI:** 10.3390/plants15040544

**Published:** 2026-02-10

**Authors:** Junda Zhang, Xinyu Wang, Yuhao Li, Zikun Yu, Ruifang Zhang, Baozhong Yin, Hongye Wang

**Affiliations:** 1 Cultivated Land Quality & Farmland Engineering Supervision and Protection Center, Ministry of Agriculture and Rural Affairs (MARA), Beijing 100020, China; jdsoil@163.com (J.Z.); wxy19951021@163.com (X.W.); talyh2016@163.com (Y.L.); yuzikun1@163.com (Z.Y.); 2 College of Land and Resources, Hebei Agricultural University, Baoding 071001, China; ruifangzhang2003@163.com; 3 College of Plant Protection, Hebei Agricultural University, Baoding 071001, China; yinbaozhong@hebau.edu.cn

**Keywords:** maize, planting density, yield, meta-analysis, Northeast China, soil organic matter

## Abstract

Increasing maize planting density is considered as a potential strategy for enhancing grain yield in Northeast China; however, its yield-enhancing effects and underlying regulatory mechanisms remain inadequately characterized. Based on 508 paired observations from 42 publications, this meta-analysis quantified the effects of increased planting density on yield components and elucidated the regulatory roles of environmental and agronomic factors. Results demonstrated that increased density intensified plant competition, raising grains per ear by 43.7% and 100-kernel weight by 6.7%. The optimal regional planting density was determined to be 89,622.6 plants ha^−1^, achieving a peak yield of 12,143 kg ha^−1^—significantly exceeding current conventional densities (49,000–65,000 plants ha^−1^) and highlighting substantial yield potential. Although nitrogen (N) management did not alter peak yield levels, it significantly increased the optimal density threshold (reaching 99,600 plants ha^−1^ under high N). Among environmental factors, mean annual precipitation (MAP) was the primary constraint: yield responses to increased density were negligible in arid regions (<400 mm); yield increase 4.0%, whereas optimal density (87,720 plants ha^−1^) and peak yield (11,747.2 kg ha^−1^) in high-rainfall regions (≥400 mm) were significantly higher than in arid regions. Soil organic matter (SOM) (>20 g kg^−1^) and optimal bulk density (BD) (1.25–1.40 g cm^−3^) synergistically enhanced optimal density and peak yield. This study confirms the substantial, yet context-dependent, yield potential of increasing planting density in Northeast China, providing a science-based framework for region-specific optimization.

## 1. Introduction

Improving food production to meet the needs of a growing global population remains a major challenge [1]. Maize (*Zea mays* L.) is one of the most widely cultivated cereal crops worldwide and serves as a staple food for over 4.5 billion people. Global maize demand is projected to double by 2050 to meet future food and feed requirements [2]. In China, where over 22% of the world’s population depends on only 9% of global arable land, increasing maize productivity per unit area is crucial to ensure food security and sustainable agricultural development [3].

In addition to genetic improvement, optimized field management, and adequate water and nutrient supply, optimizing planting density is a key agronomic practice for achieving high maize yields. The Northeast China Plain (NECP) is the largest maize production base in China, accounting for over one-third of the national maize output [4]. However, planting densities in the region typically range from 49,000 to 65,000 plants ha^−1^, which remains substantially lower than those in major producing regions like the United States (82,000–92,000 plants ha^−1^) [5]. This suggests a large potential for yield improvement through enhanced planting density and optimized canopy structure in Northeast China.

Increasing planting density can raise maize yield primarily by boosting the number of ears per unit area and improving canopy light interception and dry matter accumulation. However, high plant density intensifies competition among plants for light, water, and nutrients, which can restrict leaf expansion, reduce photosynthetic capacity, accelerate leaf senescence [6], and limit grain filling, ultimately lowering yield per plant. Within an optimal range, the reduction in per-plant yield can be compensated by the increased plant population, resulting in higher yield per unit area [7]. Beyond this threshold, however, crowding stress leads to decreased biomass partitioning to kernels and a reduction in harvest index [8]. Consequently, previous studies in China have reported positive, neutral, or negative yield responses to increasing plant density, depending on climatic conditions, genotypes, and field management practices.

In Northeast China, where characterized by a cool temperate monsoon climate, short growing seasons, limited solar radiation, and variable soil fertility interact with density management to influence maize yield responses [9]. These complex interactions make it difficult to identify a single optimal planting density for regional maize production. Moreover, independent field studies have often produced inconsistent results even under similar density increments, reflecting the influence of confounding environmental and agronomic factors. This study provides a comprehensive, quantitative synthesis specific to Northeast China, uniquely evaluating the simultaneous moderating effects of climate, soil, and management factors on maize yield responses to planting density.

Meta-analysis provides a robust approach to synthesizing results across independent studies and quantifying the overall effects and moderators influencing the outcomes [10,11]. Therefore, the present study conducted a meta-analysis based on 508 paired observations from 42 published studies to comprehensively evaluate the effects of increased maize planting density on yield and related agronomic traits in Northeast China. The specific objectives were to:

(1) Quantify the impacts of increased planting density on maize yield and yield components; and (2) Identify the key agronomic, climatic, and edaphic factors regulating yield responses to density enhancement. The findings of this study aim to provide a scientific basis for optimizing maize planting density and improving yield potential under sustainable intensification in the Northeast China Plain.

## 2. Results

### 2.1. Overall Effects of Increased Planting Densities on Maize Yield

**Figure 1:**
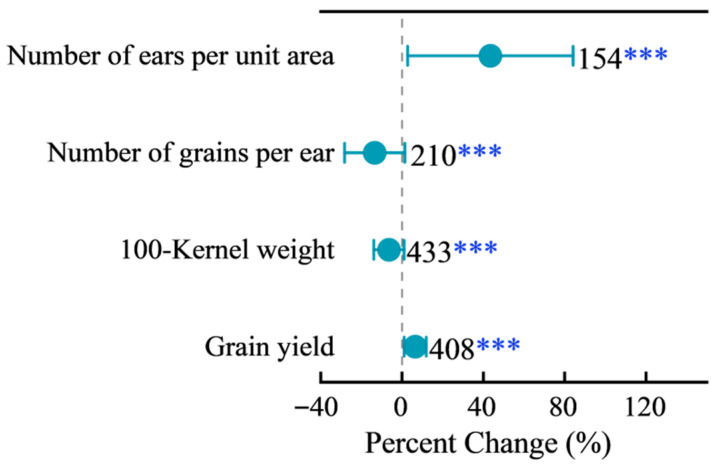
Effects of increased planting density on maize yield-related traits. Data are presented as percent changes relative to conventional planting density (CK). The closed circle indicates the mean percent change, and the horizontal line represents the 95% confidence interval. Asterisks denote significant difference from zero (*** *p* < 0.001).

Increasing planting density significantly altered maize yield components (Figure 1). It reduced the number of grains per ear by 13.3% (95% CI: −14.8% to −11.7%) and the 100-kernel weight by 6.25% (95% CI: −6.2% to −5.0%). Conversely, it increased the number of ears per unit area by 43.7% (95% CI: 40.7% to 46.7%) and the grain yield by 6.7% (95% CI: 5.4% to 8.0%). Significant heterogeneity was detected across studies (*Q_M_*(*df* = 4) = 1618.45, *p* < 0.0001), highlighting the substantial influence of moderating factors explored in subsequent sections.

### 2.2. Optimal Planting Density and Maximum Grain Yield of Maize in Northeast China

**Figure 2:**
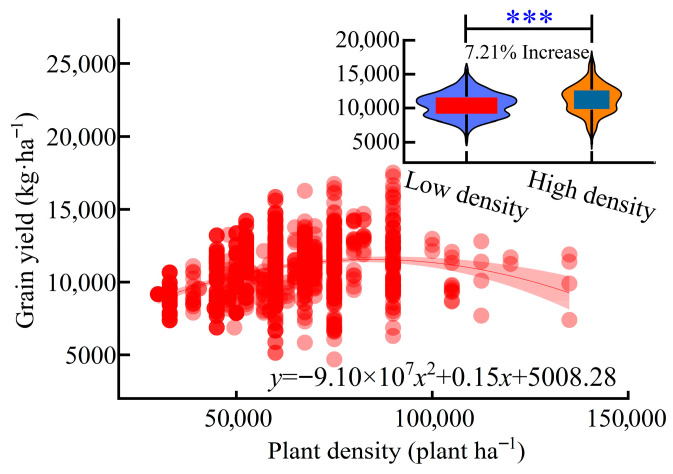
Relationship between planting density and maize grain yield in Northeast China. Scatter points represent field-observed grain yield under different planting densities (×10^4^ plants ha^−1^). (*** *p* < 0.001). The quadratic regression model shows that the red line represents the changes in yield under different planting densities; the box plot indicates the data of yield after low-density planting (blue) and the data of yield after high-density planting (orange).

Quadratic regression analysis revealed that maize grain yield followed a parabolic relationship with planting density, initially increasing and then declining. The model projected a maximum yield of 12,143.0 kg·ha^−1^ at the optimal density of 89,622.6 plants·ha^−1^. Notably, grain yield began to decline when density exceeded 12 × 10^4^ plants·ha^−1^ (Figure 2).

Furthermore, box plot analysis confirmed significantly higher grain yields under higher-density regimes (>6 × 10^4^ plants ha^−1^) compared to lower-density groups (≤6 × 10^4^ plants ha^−1^), with an 18.5% increase in median yield.

### 2.3. Effects of Nitrogen Application Rates on Maize Yield Response to Increased Planting Density

**Figure 3:**
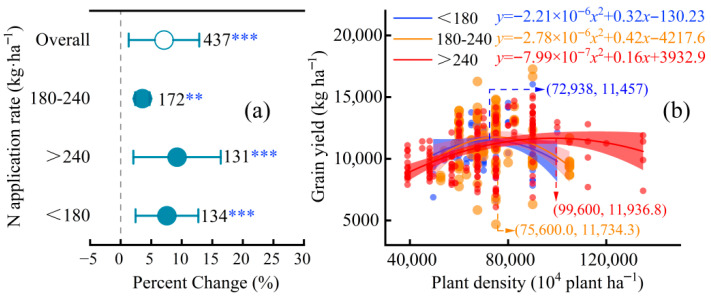
(**a**) Percent change in maize grain yield under increased planting density relative to conventional density, stratified by nitrogen (N) application rates: low (<180 kg·ha^−1^), medium (180–240 kg ha^−1^), high (>240 kg ha^−1^), and overall (all rates combined). (**b**) Quadratic regression models illustrating the relationship between planting density (plants ha^−1^) and maize grain yield (kg ha^−1^) for low (blue), medium (orange), and high (red) N application rates. (** *p* < 0.01, *** *p* < 0.001).

The yield response to increased planting density was strongly moderated by nitrogen (N) application rates (Figure 3) (*Q_M_*(*df* = 4) = 247.8535, *p* < 0.0001). Based on regional fertilization practices, N rates were categorized as low (<180 kg N ha^−1^), medium (180–240 kg N ha^−1^), and high (>240 kg N ha^−1^) [5,8]. Subgroup analysis revealed a significant yield increase only under low N conditions (+7.6%; 95% CI, 5.1–10.0%; *p* < 0.001), with non-significant responses under medium and high N.

Quadratic regression modeling further elucidated the density-yield dynamics under varying N inputs (Figure 3b). The optimum planting density (i.e., the density maximizing grain yield) increased with N availability: 72,398 plants ha^−1^ under low N, 75,600 plants ha^−1^ under medium N, and 99,600 plants ha^−1^ under high N. Notably, although the optimal density rose by approximately 38% from low to high N, the corresponding peak grain yields, the peak grain yields achieved at these optimal densities did not differ significantly among the three N-rate categories.

### 2.4. Effects of Mean Annual Temperature (MAT) and Mean Annual Precipitation (MAP) on Maize Yield Response to Increased Planting Density

**Figure 4:**
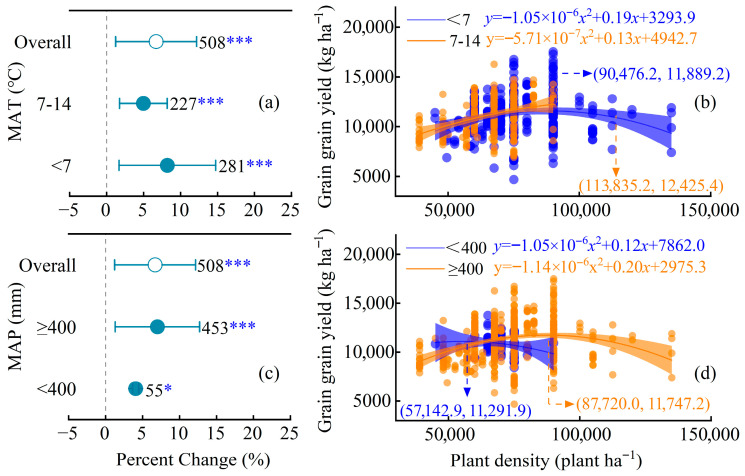
(**a**) Yield response to increased planting density across MAT groups. (**b**) Density–yield relationships for MAT groups; low MAT: <7 °C, blue circles; high MAT: 7–14 °C, orange circles. (**c**) Yield response across MAP groups. (**d**) Density–yield relationships for MAP groups; low MAP: <400 mm, blue circles; high MAT: ≥400 mm, orange circles. (* *p* < 0.05,*** *p* < 0.001).

The yield response to increased planting density was significantly moderated by mean annual precipitation (MAP) but not by mean annual temperature (MAT) (Figure 4). Overall, increasing density significantly raised yield by 6.7% (95% CI, 5.5–7.9%; *Q_M_*(*df* = 3) = 255.2571, *p* < 0.0001). When stratified by MAT, yield increases were significant in both low (<7 °C; +8.2%) and medium (7–14 °C; +5.0%) temperature groups, with no statistical difference between them (*p* > 0.05). In contrast, MAP had a pronounced effect: the yield response was negligible in arid regions (<400 mm; +4.0%, *p* > 0.05) but significant in regions with ≥400 mm precipitation (+7.0%; *p* < 0.001). Quadratic regression indicated that the optimal planting density and peak yield were substantially higher in the high-MAP region (87,720 plants ha^−1^ and 11,747 kg ha^−1^, respectively) compared to the low-MAP region (57,143 plants ha^−1^ and 11,292 kg ha^−1^) (Figure 4d).

### 2.5. Effects of Increasing the Planting Density Under Different Soil Conditions

**Figure 5:**
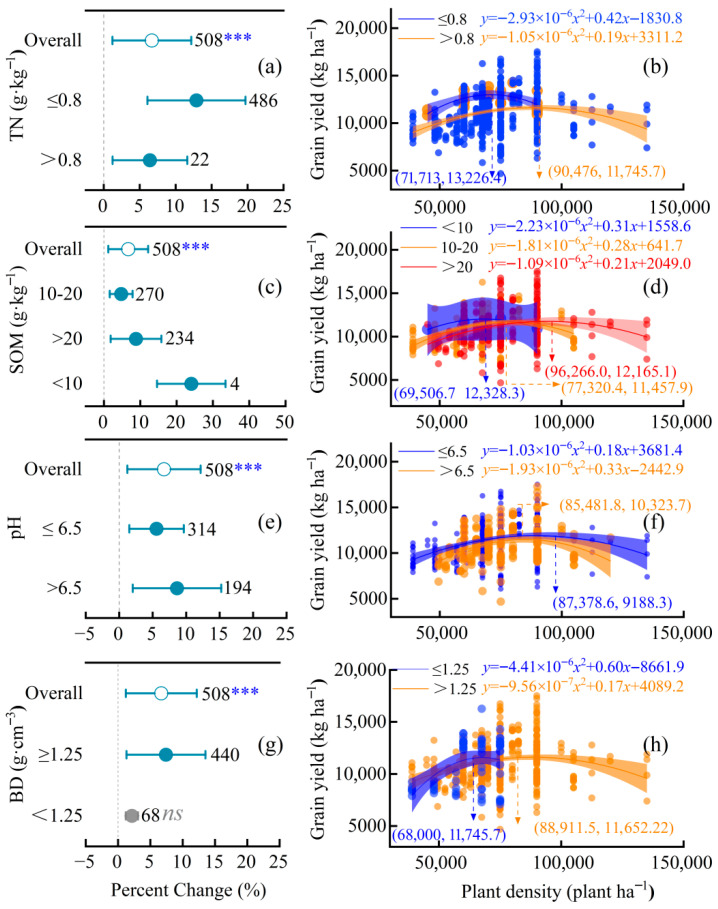
Effects of soil properties on maize yield response to increased planting density. (**a**,**c**,**e**,**g**) Percent yield change relative to conventional density, stratified by soil property: total nitrogen (TN), soil organic matter (SOM), pH, and bulk density (BD). *** *p* < 0.001; ns, not significant. (**b**,**d**,**f**,**h**) Quadratic regression of planting density versus grain yield. Data points and curves are color-coded by subgroup: (**b**) TN (low: ≤0.8 g·kg^−1^, blue circles; high: >0.8 g·kg^−1^, orange circles); (**d**) SOM (low: <10 g·kg^−1^, blue circles; medium: 10–20 g·kg^−1^, orange circles; high: >20 g·kg^−1^, red circles;) (**f**) pH (low: ≤6.5, blue circles; high: >6.5, orange circles); (**h**) BD (low: ≤1.25 g·cm^−3^, blue circles; high: >1.25 g·cm^−3^, orange circles).

All measured soil factors significantly moderated the density–yield relationship (*p* < 0.001; Figure 5). The yield increase was most pronounced under low total nitrogen (TN ≤ 0.8 g kg^−1^), reaching 12.9% (95% CI: 6.8 to 19.3%), and was significantly higher than under high TN (*p* < 0.001). Quadratic models showed the highest optimal density (90,476 plants ha^−1^) occurred at high TN, but the highest peak yield (13,226 kg ha^−1^) at low TN (Figure 5a,b).

For soil organic matter (SOM), yield response was stronger in low-SOM soils (<10 g kg^−1^), while optimal density and peak yield increased progressively with SOM content (Figure 5c,d). Although yield increased significantly across pH groups, the response was greater in acidic soils (pH ≤ 6.5), whereas optimal density was higher in neutral-to-alkaline soils (Figure 5e,f).

Critically, bulk density (BD) exhibited a clear optimum: yield response peaked within the range of 1.25–1.40 g cm^−3^ (Figure 5g,h).

### 2.6. Model-Averaged Relative Importance of Predictors in Explaining Maize Yield Responses to Increased Planting Density

**Figure 6:**
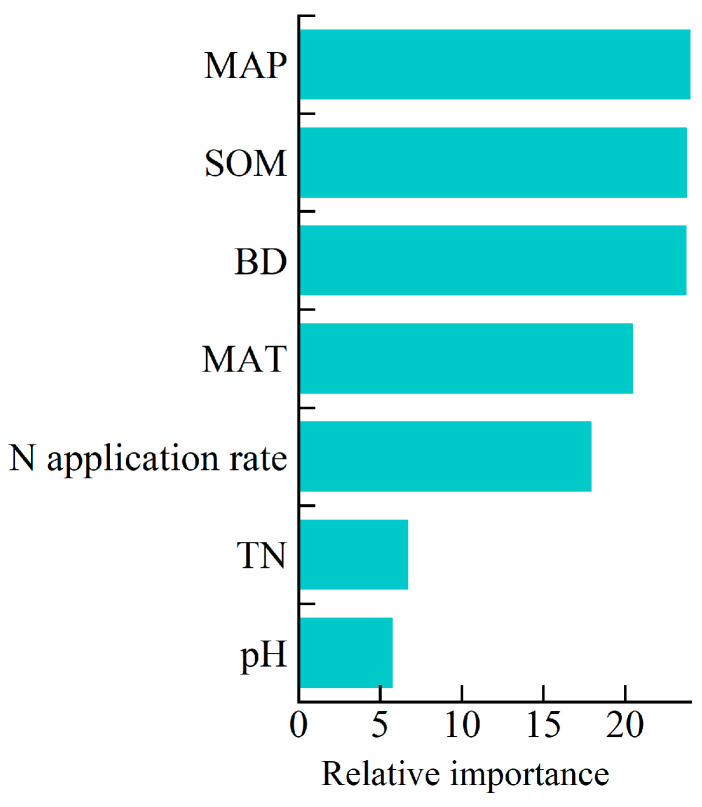
Relative importance of environmental (MAP, MAT), soil (SOM, BD, TN, pH), and management (N application) factors in explaining maize yield responses to increased planting density.

Model-averaged analysis (Figure 6) identified mean annual precipitation (MAP), soil organic matter (SOM), and bulk density (BD) as the most influential factors moderating the yield response to increased planting density. Nitrogen application rate and mean annual temperature (MAT) played moderate roles, while total soil nitrogen (TN) and pH contributed minimally.

## 3. Discussion

Our meta-analysis, synthesizing data from 508 field observations, reveals a complex and heterogeneous relationship between planting density and maize yield in Northeast China. This substantial variability (I^2^ > 80%) is not a limitation but rather a key finding: it underscores that the yield response to densification is profoundly context-dependent. The primary objective of this discussion is to decipher this heterogeneity by quantifying and interpreting the moderating roles of key agronomic, climatic, and edaphic factors. Below, we explore the trade-offs and synergies that define the optimal density window across diverse production contexts.

### 3.1. Trade-Offs Between Population-Level Gains and Individual Plant Constraints

Our meta-analysis confirms that increasing planting density enhances maize yield at the population level (+5.3%) but imposes significant constraints on individual plant growth (Figure 1). The observed reduction in grains per ear (−13.3%) and 100-kernel weight (−6.3%) aligns with established physiological principles: intensified competition for light, water, and nutrients under high density suppresses photosynthetic capacity, leaf expansion, and dry matter partitioning to kernels. In densely planted stands, individual plants experience reduced radiation interception per leaf area, leading to incomplete kernel development and lower sink strength, which may be attributed to source limitation or altered assimilate partitioning during grain filling [12].

This trade-off—where yield gains at the population scale compensate for per-plant yield penalties—represents a fundamental feature of high-density cropping systems and helps explain why density optimization, rather than maximization, is critical for productivity [7,12,13]. Increased canopy closure improves radiation interception efficiency, particularly during the early reproductive stages, yet excessive crowding accelerates leaf senescence, increases self-shading, and suppresses lower canopy photosynthesis. The yield-density relationship therefore follows a quadratic trend: yield initially rises with density due to improved light capture and resource use, then plateaus or declines when resource competition outweighs collective gains.

In Northeast China, our calculated optimal density of 89,622.6 plants ha^−1^ (yielding 12,143.0 kg ha^−1^; Figure 2) represents a 37–83% increase over current regional practices (49,000–65,000 plants ha^−1^). This substantial gap highlights untapped potential for closing yield gaps in the region. The results also suggest that many existing density recommendations may be outdated, as they were established for older, less compact cultivars. Modern hybrids with improved canopy architecture and stress tolerance can maintain higher photosynthetic efficiency and harvest index under crowding, justifying an upward revision of density thresholds. Additionally, optimizing spatial uniformity-via precision planting or mechanical adjustment-can further balance intra-row competition and enhance per-area productivity.

### 3.2. Nitrogen Management: Regulating Density Thresholds Without Altering Yield Ceilings

Changes in yield components are not driven solely by planting density in isolation, but by its interaction with key agronomic and environmental factors. The negative effects of high density on individual plants can be significantly mitigated under optimal nitrogen and water management, as demonstrated by our subgroup analyses (Figure 3, Figure 4 and Figure 5).

This study reveals a unique role of nitrogen (N) fertilization in high-density maize systems: N application rate significantly modulates optimal planting density but does not alter peak grain yield (Figure 3). Under low N inputs (<180 kg ha^−1^), the optimal density was 72,398 plants ha^−1^, while high N inputs (>240 kg ha^−1^) increased optimal density by 38% to 99,600 plants ha^−1^. Crucially, peak yields remained statistically invariant across low, medium, and high N rates (Figure 3b).

This finding indicates that N availability primarily determines the density capacity—the threshold of population tolerance to intraspecific competition—rather than the attainable yield ceiling, which remains constrained by light and hydrothermal resources [14,15]. Nitrogen promotes chlorophyll synthesis, prolongs leaf longevity, and enhances photosynthetic resilience under crowding, thereby allowing denser stands to sustain growth. However, once canopy photosynthesis reaches saturation, additional N cannot further raise yield ceilings, as grain filling becomes limited by radiation and temperature.

These results challenge the conventional paradigm that higher N inputs directly translate into yield gains. In high-density systems, N functions more as a “modulator” enabling structural adaptation and resource balance rather than as a “driver” of yield enhancement. From a management standpoint, this implies that synchronizing N availability with plant demand—through split applications or slow-release fertilizers—can stabilize yield while minimizing losses. Optimized N scheduling can also mitigate the risk of premature leaf senescence often observed in N-deficient, densely planted crops. Thus, strategic N management supports the sustainability of intensive maize systems without compromising environmental integrity.

### 3.3. Climate and Soil Mediators of Density Efficacy

Climatic regulation of maize yield responses to increased planting density exhibited marked heterogeneity, with mean annual precipitation (MAP) emerging as the primary limiting factor. In low-MAP regions (<400 mm), density increases yielded negligible yield gains (+4.0%, *p* > 0.05), whereas high-MAP areas (≥400 mm) achieved significant yield improvements (+7.0%), supported a substantial surge in optimal planting density, and elevated peak grain yield (Figure 4c,d). This divergence underscores water’s critical role in mitigating competition stress-ample precipitation ensures balanced water allocation under high-density conditions [16]. Sufficient water availability not only alleviates stomatal limitation but also stabilizes canopy temperature and facilitates nutrient uptake, leading to more synchronized ear development across plants.

Conversely, mean annual temperature (MAT) exerted no significant influence: yield responses showed no statistical differences across low (<7 °C), medium (7–14 °C), and high (>14 °C) MAT groups (3.2–9.9%; *p* > 0.05; Figure 4a). This apparent temperature resilience likely reflects maize ’s broad adaptability within the temperate monsoon climates of Northeast China. It should be noted that our use of MAT, rather than growing-season-specific metrics, may integrate both critical and non-critical thermal periods [17]. Therefore, while our analysis suggests MAT is not a primary moderator, future research employing finer temporal resolution could refine insights into temperature effects during key developmental stages. Furthermore, projected climate warming may extend the growing season and alter density optima, implying that future management strategies must integrate considerations of both cumulative heat units and water balance.

Soil properties exhibited hierarchical control over density responses, where soil organic matter (SOM) and bulk density (BD) were pivotal regulators. High-SOM soils (>20 g kg^−1^) increased optimal density by 27.8% and reduced the maximum yield by only 1.3% compared to low-SOM soils (Figure 5d). SOM buffers high-density stress by enhancing soil structure, water retention, and nutrient supply capacity, thereby promoting sustained root activity during critical growth stages. Similarly, moderate BD (1.25–1.40 g cm^−3^) optimized root-zone conditions, ensuring better root penetration and aeration. Yields under this ideal BD exceeded those in looser soils (<1.25 g cm^−3^) by 71.1% (Figure 5h), suggesting that adequate compaction supports stable anchorage and root–soil contact for efficient water use.

In contrast, total nitrogen (TN) and pH played minimal roles. Model-averaged analysis ranked TN as the least important predictor (Figure 6), and although low-TN soils (≤0.80 g kg^−1^) showed stronger relative yield responses (+12.9%), their absolute peak yields remained lower than those of medium/high-TN groups (Figure 5b). This confirms that native soil N cannot substitute for fertilizer N in regulating density thresholds, while pH’s weak effect derives from its general suitability (6–8) across the region. Overall, soil quality and water availability jointly define the physiological buffer zone within which density intensification can be successful.

### 3.4. Strategic Intensification: Synergizing Density, Nitrogen, and Soil Health

To harness the yield potential of high-density maize systems in Northeast China, three interdependent strategies emerge. First, prioritize density escalation to ≥9.0 × 10^4^ plants·ha^−1^ in high-MAP zones (>600 mm; e.g., Eastern Songnen Plain), while integrating water-saving techniques (drip irrigation, mulching) in arid areas (<400 mm) to overcome precipitation limitations [18,19,20]. Second, implement precision nitrogen management by aligning N inputs (180–240 kg ha^−1^) with target planting densities (e.g., higher N for densities > 9.0 × 10^4^ plants ha^−1^) to mitigate environmental risks without compromising yield ceilings—a critical insight given N’s role in expanding density capacity rather than elevating yield maxima [21,22]. Third, enhance soil health through residue retention and organic amendments to boost SOM (>20 g kg^−1^), coupled with subsoiling to maintain optimal BD (1.25–1.40 g cm^−3^), thereby creating resilient root environments for high-density stands.

Beyond these agronomic interventions, integrating digital farming tools-such as remote sensing, canopy imaging, and data-driven decision support-can further refine density–nutrient interactions across spatially variable fields. This precision intensification paradigm not only maximizes yield but also improves input efficiency and ecosystem resilience.

This integrated approach—density-driven intensification conditioned on water availability, precision-nutrient matching, and soil health investment—resolves the trade-off between population-level gains and individual plant constraints, unlocking sustainable yield advances in NECP maize systems [23,24,25]. It offers a practical roadmap toward climate-smart, resource-efficient maize production aligned with regional sustainability goals.

## 4. Materials and Methods

### 4.1. Data Collection

**Figure 7:**
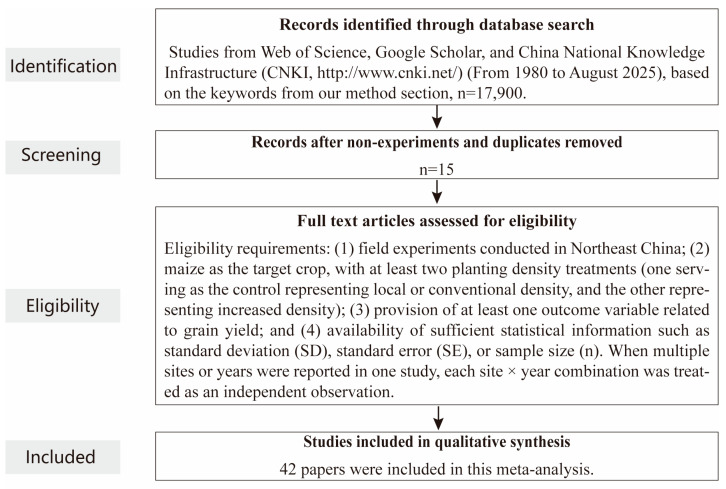
Preferred Reporting Items for Systematic Reviews and Meta-Analysis (PRISMA) flow chart illustrating the study selection procedure for the meta-analysis.

A systematic literature search was conducted to evaluate the effects of increased planting density on maize (*Zea mays* L.) grain yield in Northeast China. Following the PRISMA (Preferred Reporting Items for Systematic Reviews and Meta-Analyses) guidelines (Figure 7), peer-reviewed publications from 1990 to June 2025 were retrieved from the Web of Science, Scopus, Google Scholar, and China National Knowledge Infrastructure (CNKI) databases. The search combined English and Chinese keywords, including “maize” OR “corn”, “planting density” OR “plant population” OR “row spacing”, “grain yield”, and “Northeast China” OR “Heilongjiang” OR “Jilin” OR “Liaoning” OR “Songnen Plain” OR “Sanjiang Plain.” The search was performed in the title, abstract, and keyword fields using Boolean operators e.g., TI = (“maize”) AND AB = (“planting density”) AND AB = (“yield”). Studies were included based on the following criteria: (1) field experiments conducted in Northeast China; (2) maize as the target crop, with at least two planting density treatments (one serving as the control representing local or conventional density, and the other representing increased density); (3) provision of at least one outcome variable related to grain yield; and (4) availability of sufficient statistical information such as standard deviation (SD), standard error (SE), or sample size (n). When multiple sites or years were reported in one study, each site × year combination was treated as an independent observation. Mean annual temperature (MAT) was used as a consistent climatic proxy because specific growing-season temperature metrics were not consistently reported across the compiled studies.

From each eligible publication, the following data were extracted:

(1) General information, including author name, publication year, and study location (latitude and longitude if available); (2) experimental details, including planting density (plants ha^−1^) or row spacing (cm), cultivar type (compact or conventional), fertilizer N rate (kg ha^−1^), and management practices (e.g., irrigation, tillage); (3) environmental variables, such as soil type, mean annual temperature (MAT), and mean annual precipitation (MAP); and (4) outcome variables, including maize grain yield and yield components (if reported).

**Figure 8:**
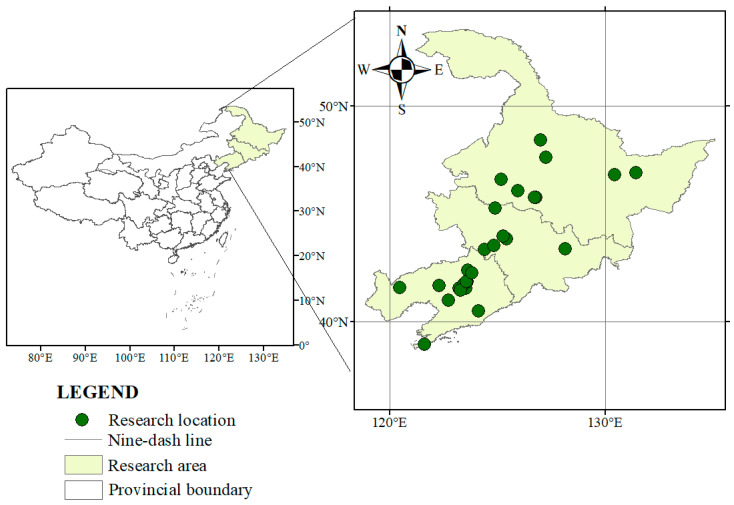
Geographic distribution of planting density experiments across 42 independent study sites included in the meta-analysis.

All data were digitized and organized using a standardized data extraction template. For studies presenting data only in graphical format, numerical values were obtained using WebPlotDigitizer (version 3.4). A total of 42 eligible publications met the inclusion criteria, yielding 508 paired observations across Northeast China. The geographic distribution of the study sites is shown in Figure 8.

Mean annual temperature (MAT) was employed as the primary thermal metric due to the consistent reporting of site-level climate normals in the source literature, whereas detailed growing-season temperature data were largely unavailable. While MAT and growing season temperature are correlated in this region, we acknowledge that MAT may not fully capture temperature effects during critical phenological stages. This limitation is considered in the interpretation of the results.

### 4.2. Meta-Analysis Procedure

To quantify how planting density and its driving factors affect yield, we used the natural-log response ratio (*lnR*) as the effect-size metric—a choice widely adopted in ecological and agronomic meta-analyses.

(1) $$lnR=ln\left(X_{t}/X_{c}\right)$$

Here, *X_t_* and *X_c_* denote the means of the treatment (increased planting density) and control (conventional density) groups, respectively.

The variance (*v*) of *lnR* for each observation was calculated as follows:

(2) $$v={{SD}_{t}}^{2}/{n_{t}X_{t}}^{2}+{{SD}_{c}}^{2}/{n_{c}X_{c}}^{2}$$

Here, *SD_t_*, *SD_c_*, *n_t_*, and *n_c_* represent the standard deviations and replicate numbers of the treatment and control groups, respectively.

The weighted effect sizes (*lnR*_++_) were determined by the following equation:

(3) $${lnR}_{++}=\sum ({lnR}_{i}{\;W}_{i})/\sum \;(W_{i})$$

Here, *lnR_i_* is the effect size of the *i*-th comparison and *W_i_* is the corresponding weight, defined as follows:

(4) $$W_{i}\left.=1/(v_{i}+\tau^{2}\right)$$

Here, *v_i_* denotes the sampling variance of *lnR_i_*, and *τ*^2^ is the between-study variance, estimated using the restricted maximum likelihood (REML) method in the rma.mv function of the R package “metafor” (version 4.5.1), implemented in R version 4.5.1.

The standard error of the weighted mean and its 95% confidence interval (CI) were calculated as follows:

(5) $$S_{lnR++}=\sqrt{1/\sum W_{i}}$$

95% CI = *lnR_++_* ± 1.96 *S_lnR_*_++_ (6)

For studies that did not report standard deviations (*SDs*), the impute_SD function in the ‘metagear’ package (R version 4.5.1) was employed to impute missing values [25]. To account for the non-independence of effect sizes arising from shared control groups, a variance–covariance matrix was constructed following the method of Lajeunesse [26].

A random-effects model was employed; this modeled variance at the study level, treatment level, and sampling error level. Parameter estimation was conducted using restricted maximum likelihood (REML). The ‘rma’ function in the ‘metafor’ package (R version 4.5.1) was used to estimate the weighted effect sizes (*lnR*_++_) and their 95% confidence intervals (CIs). An effect was deemed statistically significant if the CI did not overlap with zero. Between-group differences were considered significant if the respective CIs did not overlap [27]. Heterogeneity among studies was assessed using the Q statistic (Q_t_), with significance indicating variation in effect sizes potentially attributable to moderator variables [28]. Subgroup analyses were conducted for categorical moderators with at least ten observations or at least five observations from two or more independent studies [29]. Meta-regression analyses were conducted using the “rma()” function with the restricted maximum-likelihood estimator (REML) in the “metafor” package (R version 4.5.1) to examine the relationships between effect sizes and climatic variables, including mean annual precipitation (MAP) and mean annual temperature (MAT) [30,31].

To enhance interpretability, effect sizes were transformed into percentage changes using the following equation:

(7) $$Percent\;change=\left({exp}^{lnR}-1\right)\times 100\%$$

Using the “randomForest” package in R software version 4.2.2, a random forest model was developed to quantify the relative importance of environmental, soil, and management factors in explaining maize yield responses to increased planting density. The model incorporated environmental variables (mean annual precipitation, MAP; mean annual temperature, MAT), soil properties (soil organic matter, SOM; bulk density, BD; total nitrogen, TN; and pH), and management factors (nitrogen application rate). Statistical significance of variable importance was evaluated using the “rfPermute” package, with significance accepted at *p* < 0.05, indicating robust model performance. The relative importance of each factor was ranked based on the percentage increase in mean squared error (MSE) when that variable was permuted, averaged over 500 iterations of the random forest model.

Heterogeneity and publication bias were assessed. The random-effects model indicated substantial heterogeneity among effect sizes (I^2^ = 82.97%, *τ*^2^ = 0.0061), which was anticipated given the context-dependent nature of density effects. The subgroup and meta-regression analyses were performed to elucidate the sources of this heterogeneity. Egger’s regression test found no significant publication bias (*p* = 0.15).

## 5. Conclusions

This meta-analysis demonstrates that increasing planting density can substantially enhance maize yields in Northeast China, but with a clear optimum. Mean annual precipitation (MAP), soil organic matter (SOM), and bulk density (BD) were identified as the primary limiting factors. In contrast, nitrogen (N) management primarily regulates the tolerable plant density threshold rather than the maximum achievable yield. For regions with ≥400 mm annual precipitation, we recommend a target density of approximately 87,700 plants ha^−1^ coupled with an N application rate of 180–240 kg N ha^−1^. Combine this with organic amendments and occasional deep tillage to lift soil organic matter above 20 g kg^−1^ and keep bulk density between 1.25 and 1.40 g cm^−3^. Such an integrated package can unlock the region’s untapped yield potential while keeping production sustainable.

## Data Availability

Data is contained within the article.
